# Stratification of Individual Symptoms of Contact Lens–Associated Dry Eye Using the iPhone App DryEyeRhythm: Crowdsourced Cross-Sectional Study

**DOI:** 10.2196/18996

**Published:** 2020-06-26

**Authors:** Takenori Inomata, Masahiro Nakamura, Masao Iwagami, Akie Midorikawa-Inomata, Jaemyoung Sung, Keiichi Fujimoto, Yuichi Okumura, Atsuko Eguchi, Nanami Iwata, Maria Miura, Kenta Fujio, Ken Nagino, Satoshi Hori, Kazuo Tsubota, Reza Dana, Akira Murakami

**Affiliations:** 1 Department of Ophthalmology Juntendo University Faculty of Medicine Tokyo Japan; 2 Department of Strategic Department of Operating Room Management and Improvement Juntendo University Faculty of Medicine Tokyo Japan; 3 Department of Hospital Administration Juntendo University Graduate School of Medicine Tokyo Japan; 4 Department of Bioengineering, Precision Health Graduate School of Engineering The University of Tokyo Tokyo Japan; 5 Department of Health Services Research Faculty of Medicine University of Tsukuba Ibaraki Japan; 6 Morsani College of Medicine University of South Florida Tampa, FL United States; 7 Department of Ophthalmology Juntendo University Graduate School of Medicine Tokyo Japan; 8 Department of Electric Medical Intelligence Management Juntendo University Faculty of Medicine Tokyo Japan; 9 Department of Ophthalmology Keio University School of Medicine Tokyo Japan; 10 Department of Ophthalmology Massachusetts Eye and Ear Infirmary Harvard Medical School Boston, MA United States

**Keywords:** contact lens-associated dry eye, mobile health, ResearchKit, smartphone app, DryEyeRhythm, subjective symptoms, risk factors, dry eye, stratification, mobile phone

## Abstract

**Background:**

Discontinuation of contact lens use is mainly caused by contact lens–associated dry eye. It is crucial to delineate contact lens–associated dry eye's multifaceted nature to tailor treatment to each patient’s individual needs for future personalized medicine.

**Objective:**

This paper aims to quantify and stratify individual subjective symptoms of contact lens–associated dry eye and clarify its risk factors for future personalized medicine using the smartphone app DryEyeRhythm (Juntendo University).

**Methods:**

This cross-sectional study included iPhone (Apple Inc) users in Japan who downloaded DryEyeRhythm. DryEyeRhythm was used to collect medical big data related to contact lens–associated dry eye between November 2016 and January 2018. The main outcome measure was the incidence of contact lens–associated dry eye. Univariate and multivariate adjusted odds ratios of risk factors for contact lens–associated dry eye were determined by logistic regression analyses. The t-distributed Stochastic Neighbor Embedding algorithm was used to depict the stratification of subjective symptoms of contact lens–associated dry eye.

**Results:**

The records of 4454 individuals (median age 27.9 years, SD 12.6), including 2972 female participants (66.73%), who completed all surveys were included in this study. Among the included participants, 1844 (41.40%) were using contact lenses, and among those who used contact lenses, 1447 (78.47%) had contact lens–associated dry eye. Multivariate adjusted odds ratios of risk factors for contact lens–associated dry eye were as follows: younger age, 0.98 (95% CI 0.96-0.99); female sex, 1.53 (95% CI 1.05-2.24); hay fever, 1.38 (95% CI 1.10-1.74); mental illness other than depression or schizophrenia, 2.51 (95% CI 1.13-5.57); past diagnosis of dry eye, 2.21 (95% CI 1.63-2.99); extended screen exposure time >8 hours, 1.61 (95% CI 1.13-2.28); and smoking, 2.07 (95% CI 1.49-2.88). The t-distributed Stochastic Neighbor Embedding analysis visualized and stratified 14 groups based on the subjective symptoms of contact lens–associated dry eye.

**Conclusions:**

This study identified and stratified individuals with contact lens–associated dry eye and its risk factors. Data on subjective symptoms of contact lens–associated dry eye could be used for prospective prevention of contact lens–associated dry eye progression.

## Introduction

Contact lens (CL) wear is an established and efficient method for improving vision quality by correcting refractive errors. More than 140 million estimated CL users exist worldwide [1]. However, despite available CL products on the market, studies show that 12% to 58% of CL users discontinue CL use due to CL discomfort (CLD) [1-6]. Additionally, many continue using CLs while feeling CLD.

Dry eye disease (DED) is characterized by a tear film disorder, potentially causing ocular surface damage and ocular discomfort [7,8]. DED is becoming more prevalent due to aging society and increased digital device usage [9-13]. Accumulating studies indicate that dryness is one of the main reasons for CLD [2,3,8]; 68% to 79% of CL users have reported feeling dryness [14-16], and CL use especially is a risk factor for severe DED [17,18]. CL-associated dry eye (CLADE) may be caused by changes of tear film stability on the CL surface, decrease in the tear exchange rate, decrease in reflex secretion due to perception decline, oxygen deprivation, lens deposits, and adverse reactions to CL solutions [5]. Suggested CLADE risk factors, including environmental and host factors, and lifestyle habits are related [1,19-21]; thus, a comprehensive, multidisciplinary mass customization is needed in individual CLADE treatments. Accordingly, it is crucial to understand CLADE's multifaceted nature by monitoring various symptoms and visualizing patient lifestyle practices to improve the CL users' quality of vision through personalized treatment [22,23]. Notably, a smartphone app could effectively monitor subjective symptoms and lifestyle habits, check changes in each factor's contribution, and visualize each individual’s lifestyle [22]. The collected medical data could help lay the foundation for understanding how individual factors contribute to the aggravation of CLADE.

To reveal and simplify how multiple factors intertwine to affect CLADE's progression, we conducted this large-scale crowdsourced study using the DryEyeRhythm app (Juntendo University) to quantify and stratify the symptoms of CLADE and collect evidence for prospective prevention of CLADE progression.

## Methods

### Study Enrollment and Participants

The DryEyeRhythm app's development and the study's enrollment process have been previously described [18,24,25]. Briefly, DryEyeRhythm was developed using Apple Inc’s open-source framework, ResearchKit. DryEyeRhythm was released on Apple’s App Store in Japan on November 2, 2016, and in the United States in April 2018 [18]. Prospective participants can download the app using their own App Store credentials. This large-scale, crowdsourced, prospective, cross-sectional observational study was conducted between November 2, 2016, and January 12, 2018. All users provided electronic informed consent for participation following explanation of the study's nature and possible consequences. Duplicate users, foreign participants (outside of Japan), and users who did not complete all surveys were excluded. This study was approved by the Independent Ethics Committee of Juntendo University Faculty of Medicine (approval number 19-226) and adhered to the tenets of the Declaration of Helsinki. The methodology and results of this survey are reported according to the checklist for reporting results of internet e-surveys [26].

### User Data Collection

Using DryEyeRhythm, data on participant demographics, medical history, lifestyle habits, daily subjective symptoms, Ocular Surface Disease Index (OSDI) scores (Allergan), and Zung Self-Rating Depression Scale (SDS) scores were collected [27-29]. Multimedia Appendices 1 and 2 show specific questions and parameters used for data collection. Data that were recorded included basic demographic characteristics, including age, sex, height, body weight, race, and geographic location; medical history of hypertension, diabetes, systemic disease, mental illness, past diagnosis of DED, hay fever, and ophthalmic surgeries; and lifestyle habits, including daily coffee intake, CL information (such as the type of CL, period of CL use, and daily duration of CL use), eye drop use, screen exposure time, periodic exercise, sleep duration, smoking, and hydration. Daily subjective symptoms included eye itching, asthenopia, headache, mental fatigue, stiffness, and stress.

### Classification of CLADE

Participants were divided into the following 2 groups: non-CLADE and CLADE. Those who reported current use of CL and had an OSDI total score <13 were included in the non-CLADE group. Those who reported current use of CL and had an OSDI total score ≥13 were included in the CLADE group.

The OSDI questionnaire is a 12-item questionnaire used to assess DED severity based on ocular symptoms, impact on visual functioning, and environmental triggers [27,29]. The overall OSDI total score was determined based on a 100-point scale correlated with the severity of symptoms [30]. We previously demonstrated that the Japanese version of OSDI with DryEyeRhythm had good validity compared with that with the paper-based questionnaire [18,29,31].

Depressive symptoms were evaluated using SDS [28]. The SDS is an internationally used 20-item self-administered depression scale and has been validated in Japan. Each item is rated on a 4-point Likert scale, with a total score ranging from 20 to 80. An SDS score of ≥40 is possibly suggestive of depression [32,33].

### Statistical Analysis

Continuous variables (not normally distributed based on Shapiro-Wilk tests) are presented as medians (with interquartile ranges), and categorical variables are presented as percentages. We conducted Mann-Whitney U tests for continuous variables not normally distributed and chi-square tests for categorical variables. A comparison between negative, current, and past CL use groups was performed by one-way analysis of variance using a Bonferroni post hoc test. The odds ratio of each risk factor for CLADE was determined by multivariate adjusted logistic regression analysis, which included factors significantly associated with CLADE, as indicated by the univariable logistic regression analyses with a threshold 2-tailed, unpaired *P* value of .05. Pearson’s rank correlation coefficients were calculated to determine the correlation between each subjective symptom and CLADE. A heatmap was then made using the heatmap function of the seaborn module (version 0.9.0; Python 3). A t-distributed Stochastic Neighbor Embedding (t-SNE) was performed with a scikit-learn Python package (version 0.21.3; Python 3) [34]. *P* values were considered statistically significant at *P*<.05, *P*<.01, or *P*<.001. All data were analyzed with Stata (version 15; Stata Corp) software.

## Results

### Application Downloads and Clinical Study Enrollment

**Figure 1:**
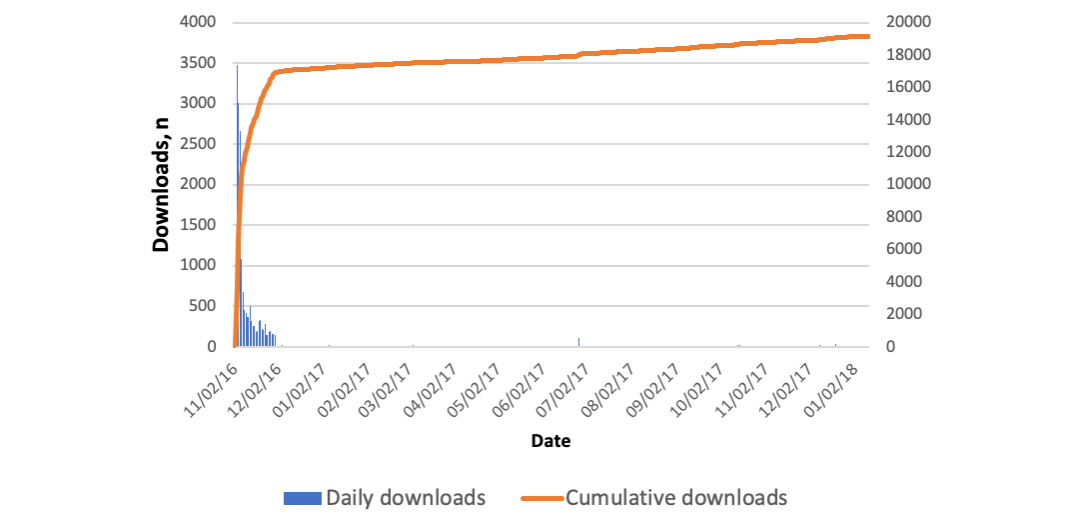
Trends in the number of downloads.

**Figure 2:**
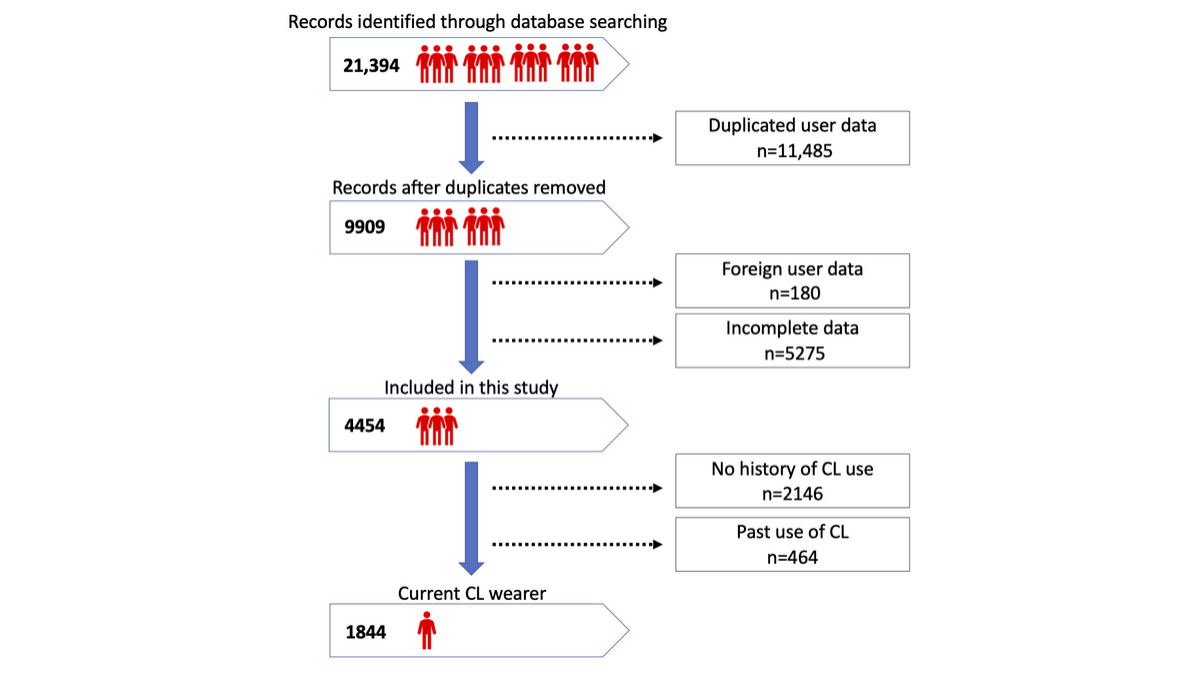
Flow chart of the enrollment process used in this study. CL: contact lens.

As seen in Figure 1, DryEyeRhythm was cumulatively downloaded 18,991 times between November 2, 2016, and January 12, 2018. As Figure 2 shows, a total of 21,394 records were identified in our crowd database; 11,485 and 5455 records were excluded from the study because of duplicate user data and incomplete survey responses, respectively. Finally, 4454 out of 9909 participants (44.95%) completed the survey and were included in the final analysis. Multimedia Appendix 3 shows the sensitivity analysis between included and excluded participants.

### Participant Characteristics

Multimedia Appendix 4 summarizes participants’ demographics, medical history, and lifestyle habits according to CL usage. The included participants' median age was 23 years (IQR 18-35), and women accounted for 66.73% (2972/4454) of the participants. Most participants were in the age group of 18 to 34 years (2362/4454 participants, 53.03%), followed by the age group of 35 to 64 years (1133/4454 participants, 25.44%). CL users were divided into the following subgroups: negative (2146/4454 participants, 48.18%), past use (464/4454 participants, 10.42%), and current use (1844/4454 participants, 41.40%). Most of the current CL users were younger than 65 years.

Regarding the medical history survey, the majority of the participants (2747/4454, 61.67%) did not present medicated hypertension, diabetes, systemic diseases, or mental illness history. Hay fever was present in about half of the participants (2249/4454, 50.49%). Previous DED diagnoses were reported in 26.08% of past (178/464) and current (481/1844) CL users, while 17.99% (386/2146 out of participants) of negative contact lens users had previous DED diagnoses. The history of laser-assisted in situ keratomileusis surgery was significantly increased in past CL users (*P*<.001).

Regarding lifestyle habits, eye drop use was reported in 23.49% (109/464) of past CL users and 26.03% (480/1844) of current CL users, while 13.42% (288/2146) of negative contact lens users did not use eye drops. Median screen exposure time was 6 hours (IQR 4-10). About two-thirds of participants (2919/4454, 65.54%) showed periodic exercise habits, with an average exercise time of 1 hour (IQR 0-3) per week. The median sleeping time was 7 hours (6-8.5) per night; however, more than half of participants (2355/4454, 52.87%) slept fewer than 6 hours per night. About a quarter (1058/4454, 23.75%) of participants smoked.

### Characteristics of Non-CLADE and CLADE Groups

Multimedia Appendix 5 shows the characteristics of non-CLADE and CLADE groups. Most participants in both non-CLADE and CLADE groups were in the 18 to 34 years age group. There were significantly more female participants in the CLADE group (*P*<.001). Height and body weight were higher in the non-CLADE group. The median CL term was 6 hours (IQR 3-10) per day in both the non-CLADE and CLADE groups, and approximately half of the participants used CL for 12 to 18 hours per day. More than 90% (1725/1844, 93.55%) of the participants in both non-CLADE and CLADE groups used soft contact lenses (SCL). The majority of the SCL types were the daily disposable type (823/1844 participants, 44.63%), followed by the biweekly (every 2 weeks) type (741/1844 participants, 40.18%). Regarding the medical history survey, the prevalence of hay fever (*P*=.002), past diagnosis of DED (*P*<.001), and mental illnesses other than depression or schizophrenia (*P*=.003) were significantly higher in the CLADE group than in the non-CLADE group. Regarding lifestyle habits, eye drop use (*P*<.001), screen exposure time (*P*<.001), and smoking habits (*P*=.001) were significantly higher in the CLADE group than in the non-CLADE group.

### Subjective Symptoms

Table 1 shows daily subjective symptoms and OSDI and SDS data. Eye itching was significantly higher in the CLADE group than in the non-CLADE group (*P*<.001). Other subjective symptoms, including asthenopia, headache, mental fatigue, and stiffness, were higher in the CLADE group compared with the non-CLADE group. The median OSDI total scores were 8.3 (IQR 6.3-10.4) in the non-CLADE group and 30 (IQR 20.8-43.8) in the CLADE group. Subscale scores of OSDI were higher in the CLADE group than in the non-CLADE group. The SDS score was also higher in the CLADE group, and 80.10% (1159/1447) of participants in this group experienced depressive symptoms (SDS score ≥40).

**Table 1:** Subjective symptoms of contact lens–associated dry eye.

Daily subjective symptoms			Non-CLADE^a^ (n=397)		CLADE (n=1447)		*P* value	
Eye itching (0-10), median (IQR)			0 (0-2)		2 (0-5)		<.001	
Asthenopia (yes), n (%)			147 (37.0)		899 (62.1)		<.001	
Headache (0-10), median (IQR)			0 (0-2)		1 (0-4)		<.001	
Mental fatigue (yes), n (%)			57 (14.4)		446 (30.8)		<.001	
Stiffness and pain of body axis muscle (yes), n (%)			151 (38.0)		816 (56.4)		<.001	
Stress (1-10), median (IQR)			4 (2-6)		5 (3-7)		<.001	
J-OSDI^b^ total score (1-100), median (IQR)			8.3 (6.3-10.4)		30 (20.8-43.8)		<.001	
**Ocular symptoms (0-100), median (IQR)**			15 (5-20)		35 (25-45)		<.001	
	(1) Eyes that are sensitive to light (0-4), median (IQR)			1 (0-1)		1 (1-3)		<.001
	(2) Eyes that feel gritty (0-4), median (IQR)			1 (0-1)		1 (1-2)		<.001
	(3)Painful or sore eyes (0-4), median (IQR)			0 (0-1)		1 (1-2)		<.001
	(4) Blurred vision (0-4), median (IQR)			0 (0-1)		1 (1-2)		<.001
	(5) Poor vision (0-4), median (IQR)			0 (0-1)		1 (1-2)		<.001
**Vision-related function (0-100), median (IQR)**			0 (0)		12.5 (6.3-25)		<.001	
	(6) Reading (0-4), median (IQR)		0 (0)		1 (0-1)		<.001	
	(7) Driving at night (0-4), median (IQR)		0 (0)		0 (0-1)		<.001	
	(8) Working with a computer or bank machine (0-4), median (IQR)		0 (0)		0 (0-1)		<.001	
	(9) Watching TV (0-4), median (IQR)		0 (0)		1 (0-1)		<.001	
**Environmental triggers (0-100), median (IQR)**			8.3 (8.3-16.7)		41.7 (25-66.7)		<.001	
	(10) Windy conditions (0-4), median (IQR)		0 (0)		1 (1-3)		<.001	
	(11) Places or areas with low humidity (0-4), median (IQR)		0 (0-1)		2 (1-3)		<.001	
	(12) Areas that are air conditioned (0-4), median (IQR)		0 (0-1)		2 (1-3)		<.001	
SDS^c^ score (20-80), median (IQR)			42 (36-49)		48 (41-55)		<.001	
SDS score >40, n (%)			234 (58.9)		1159 (80.1)		<.001	

^a^CLADE: contact lens–associated dry eye.

^b^J-OSDI: Japanese Ocular Surface Disease Index.

^c^SDS: Zung Self-Rating Depression Scale.

### Stratification of Subjective Symptoms of CLADE

**Figure 3:**
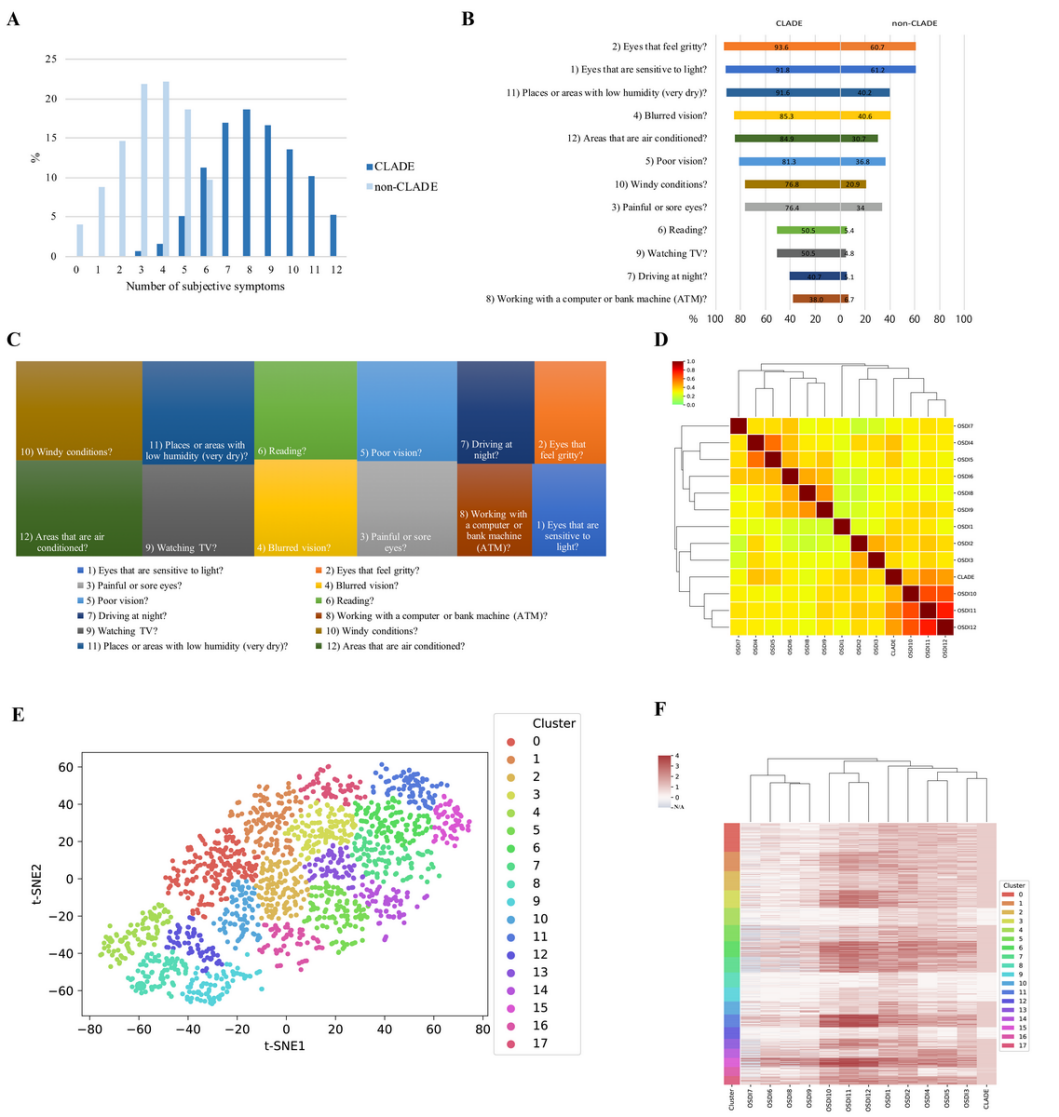
Stratification of subjective symptoms of CLADE. (A) The number of subjective symptoms in the non-CLADE and CLADE groups. (B) The frequency of subjective symptoms of DED based on each OSDI questionnaire. (C) The treemap shows the difference in the frequency of subjective symptoms of DED (the percentage of subjective symptoms in the CLADE group minus the percentage of subjective symptoms in the non-CLADE group) based on each OSDI question. (D) The Pearson correlation coefficients between subjective symptoms and CLADE are shown in the heatmap as a color gradient. (E) t-SNE projection shows the groups of CLADE based on the subjective symptoms. (F) The heatmap shows the correlation between the groups made by t-SNE projection and each OSDI item. CLADE: contact lens–associated dry eye. DED: dry eye disease. OSDI: Ocular Surface Disease Index. t-SNE: t-distributed Stochastic Neighbor Embedding.

Figure 3 shows various representations of the stratification of the subjective symptoms of CLADE. The number of subjective symptoms for both CLADE and non-CLADE groups is based on OSDI questionnaires, and the number of subjective symptoms was higher in the CLADE group. The rate of positive signs in each OSDI item between the CLADE and non-CLADE groups shows that over 90% of CLADE participants felt gritty eyes, were sensitive to light, and felt uncomfortable in low-humidity places. The treemap consists of the differences between the CLADE and non-CLADE groups in the frequency of subjective symptoms of DED triggered by environmental factors (OSDI questions 10-12). The environmental factors are characterized as subjective symptoms of CLADE. The heatmap that visualizes the correlation between each subjective symptom and CLADE demonstrates that the subjective symptoms triggered by environmental factors (OSDI questions 10-12) were highly positively correlated with CLADE. The t-SNE projection of CLADE and non-CLADE groups according to the subjective symptoms shows that the CLADE group had a variety of subjective symptoms compared with the non-CLADE group (14 groups vs 4 groups, respectively). Finally, we created a heatmap in which the patterns in the 18 groups based on each subjective symptom were stratified by the t-SNE projection. Those 18 groups were subgrouped based on the OSDI as follows: ocular symptoms (OSDI questions 1-5), vision-related function (questions 6-9), and environmental triggers (questions 10-12).

### Risk Factors for CLADE

Table 2 shows univariate odds ratios of each risk factor for CLADE, determined by logistic regression analysis. The univariate odds ratios were 0.99 (95% CI 0.975-0.998) for age, 1.75 (95% CI 1.33-2.23) for female sex, 0.98 (95% CI 0.97-0.99) for height, 1.41 (95% CI 1.13-1.77) for hay fever, 3.13 (95% CI 1.43-6.84) for mental illness other than depression or schizophrenia, 2.11 (95% CI 1.58-2.82) for past diagnosis of DED, 1.56 (95% CI 1.11-2.19) for more than 8 hours of screen exposure per day, and 1.69 (95% CI 1.26-2.28) for smoking. Relevant risk factors for CLADE were identified by the analyses of univariate odds ratios and were further analyzed by multivariate analyses. Table 2 also shows the multivariate odds ratios of each risk factor for CLADE. The multivariate odds ratios were 0.98 (95% CI 0.96-0.99) for age, 1.53 (95% CI 1.05-2.24) for female sex, 1.38 (95% CI 1.10-1.74) for hay fever, 2.51 (95% CI 1.13-5.57) for mental disease other than depression or schizophrenia, 2.21 (95% CI 1.63-2.99) for past diagnosis of DED, 1.61 (95% CI 1.13-2.28) for more than 8 hours of screen exposure per day, and 2.07 (95% CI 1.49-2.88) for smoking. Number of years of CL use, duration worn per day, and CL type had no significant association with CLADE.

**Table 2:** Univariate and multivariate adjusted odds ratios of risk factors for contact lens–associated dry eye.

Risk factors						Univariate odds ratio (95% CI)		Multivariate odds ratio (95% CI)	
**Demographic characteristics**									
	Age (per year)					0.99 (0.975-0.998)		0.98 (0.96-0.99)	
	Sex (female vs male)					1.75 (1.33-2.23)		1.53 (1.05-2.24)	
	Height (cm)					0.98 (0.97-0.99)		1.00 (0.98-1.02)	
	Weight (kg)					0.99 (0.98-1.00)		N/A^a^	
CL^b^ use (0-10 years)						0.98 (0.95-1.02)		0.99 (0.95-1.03)	
**CL duration (hours per day)**									
	0-6					1 (N/A)		1 (N/A)	
	7-12					1.25 (0.77-2.05)		1.28 (0.76-2.14)	
	13-18					1.19 (0.74-1.92)		1.24 (0.74-2.06)	
	19-24					1.77 (0.93-3.36)		1.45 (0.73-2.85)	
**Contact lens type**									
	Hard					1 (N/A)		1 (N/A)	
	Disposable (daily)					0.98 (0.61-1.58)		0.82 (0.49-1.36)	
	Disposable (biweekly^c^)					0.87 (0.54-1.40)		0.76 (0.46-1.28)	
	Disposable (monthly)					1.30 (0.71-2.39)		0.92 (0.48-1.77)	
	Conventional lens					1.23 (0.63-2.39)		0.90 (0.44-1.83)	
Medicated hypertension (yes vs no)						0.51 (0.23-1.16)		N/A	
Diabetes (yes vs no)						0.96 (0.20-4.64)		N/A	
**Systemic diseases (yes** **vs** **no)**									
	Blood disease					1.92 (0.24-15.69)		N/A	
	Brain disease					0.68 (0.13-3.54)		N/A	
	Collagen disease					2.20 (0.27-17.65)		N/A	
	Heart disease					2.12 (0.63-7.10)		N/A	
	Kidney disease					2.91 (0.68-12.50)		N/A	
	Liver disease					1.51 (0.33-6.85)		N/A	
	Malignant tumor					N/A		N/A	
	Respiratory disease					1.62 (0.96-2.75)		N/A	
Hay fever (yes vs no)						1.41 (1.13-1.77)		1.38 (1.10-1.74)	
**Mental illness (yes** **vs** **no)**									
	Depression						1.91 (0.86-4.26)		N/A
	Schizophrenia						1.01 (0.28-3.62)		N/A
	Other						3.13 (1.43-6.84)		2.51 (1.13-5.57)
Past diagnosis of dry eye disease						2.11 (1.58-2.82)		2.21 (1.63-2.99)	
**Ophthalmic surgery (yes** **vs** **no)**									
	Cataract surgery						N/A		N/A
	LASIK^d^						0.27 (0.04-1.95)		N/A
	Other						2.58 (0.91-7.28)		N/A
Coffee (per cup per day)						1.00 (0.92-1.10)		N/A	
**Screen exposure time (hours)**									
	<4						1 (N/A)		1 (N/A)
	4-8						1.08 (0.80-1.45)		1.07 (0.79-1.45)
	>8						1.56 (1.11-2.19)		1.61 (1.13-2.28)
Periodic exercise (yes vs no)						0.83 (0.66-1.05)		N/A	
**Sleeping time (per hour per day)**									
	<6					1.05 (0.82-1.36)		N/A	
	6-9					1 (N/A)		N/A	
	>9					1.24 (0.91-1.68)		N/A	
Smoking (yes vs no)						1.69 (1.26-2.28)		2.07 (1.49-2.88)	
Water intake (per 100 mL/d)						1.00 (1.00-1.00)		N/A	

^a^N/A: not applicable.

^b^CL: contact lens.

^c^Biweekly: every 2 weeks.

^d^LASIK: laser-assisted in situ keratomileusis.

## Discussion

CL use significantly contributes to the quality of vision by correcting refractive errors. However, many CL users discontinue CL wear because of CLD [1-6]. This study analyzed individuals’ medical data obtained from the DryEyeRhythm app and clarified the characteristics of subjective symptoms of CLADE and its risk factors. We found that CLADE remained undiagnosed in many individuals who experienced dry eye symptoms and had not been treated with eye drops. Since CLADE is a risk factor for CL discontinuation, evaluating individual subjective symptoms of CLADE and identifying personalized and preemptive medical care will contribute to more comfortable CL use. This study found that younger age, female sex, hay fever, mental illness other than depression or schizophrenia, extended screen exposure time, and smoking were CLADE risk factors. Our findings might help develop individual preemptive strategies for CLADE.

New medical big data collected from mobile health apps and the Internet of Medical Things have been used in recent years [18,22,35-37]. Because CL users are relatively young [38,39], using innovative methods such as smartphone apps is crucial in investigating individual subjective symptoms. This app recruitment model is more inclusive of younger people because they are relatively healthy and seldom visit hospitals. Indeed, many younger individuals (median age of 23 years) participated in this crowdsourced study due to DryEyeRhythm. Previous studies presented challenges regarding information collection for individuals who previously wore CL [1]; however, this crowdsourced study allowed an easier collection of the information compared with the conventional hospital-based study. This study found that 41.40% (1844/4454) of current CL users and 10.42% (464/4454) of past CL users discontinued CL use for various reasons, as Multimedia Appendix 4 showed. Moreover, as demonstrated in previous studies [40], the proportion of current CL users tended to decrease with increasing age, as seen in Multimedia Media Appendix 4. Our findings are consistent with those of previous studies using hospital-based, mail-based, email-based, and Facebook-based methods, which found that between 12% and 51% of CL users discontinued use [2,3,6]. Therefore, this mobile-based health study can be used to supplement traditional hospital-based studies.

Because 23% of the symptomatic participants did not exhibit typical clinical signs of dryness [41], investigating subjective symptoms of CLADE is likely to have more diagnostic value than conducting clinical tests. In this study, 78.47% (1447/1844) of current CL users had CLADE with an OSDI score ≥13. Given that only 28.82% (417/1447) of participants with CLADE had been diagnosed with DED in the past and only 27.92% (404/1447) of participants with CLADE had used eye drops, this study could assess the proportion of individuals who were undiagnosed with DED and did not undergo treatment intervention while experiencing CLADE symptoms. However, since CLADE presents various subjective symptoms, it is possible that those CL users might already have been experiencing CLADE symptoms but were not diagnosed. This study found that individuals with CLADE had multiple subjective symptoms, and more than 90% of participants with CLADE reported that their eyes felt gritty (1355/1447 participants, 93.64%) and were sensitive to light (1328/1447 participants, 91.78%). In particular, items related to environmental triggers were more frequent in CLADE patients than in non-CLADE patients (Figure 3), indicating that CLADE may be strongly influenced by environmental factors. Therefore, improvement of environmental triggers may be a potential intervention method to prevent CL discontinuation due to CLADE. Furthermore, this study stratified various individual subjective symptoms of CLADE using a multidimensional analysis with t-SNE (Figure 3), and the subjective symptoms of CLADE were divided into 14 subgroups. Some CLADE subgroups were strongly related to environmental factors and others were not. The findings indicate that it is important to conduct personalized medicine based on individual CLADE symptoms.

Our study aimed to identify risk factors that contribute to CLADE in a large-scale prospective clinical study using real-world data. The resulting data are shown as odds ratios of risk factors for CLADE (Table 2), including younger age, female sex, hay fever, mental illness other than depression or schizophrenia, past diagnosis of DED, extended screen exposure time, and smoking. Among these risk factors, female sex, mental illness other than depression or schizophrenia, extended screen exposure time, and smoking are associated with DED [17]. Young age is also a risk factor for CLADE, probably because of the higher sensitivity to CLADE symptoms among CL users in the younger cohort and the cessation of CL use in the older cohort due to DED [11,24]. Our study revealed that CLADE was recognized by many young CL users, suggesting the importance of the prevention and treatment of CLADE among young CL users. It should be noted that many CL users were women (1471/1844, 79.77%), and although our results do not directly associate the physiology of female sex with the pathology of CLADE, we believe that the significantly higher prevalence of CL use in the female cohort warrants the recognition of the female population as a relative risk factor for CLADE and DED. This study showed that the median wearing time per day of CL was 14 hours (IQR 12-16), indicating that CL are worn almost all day. We also demonstrated that over 8 hours of screen exposure time was a risk factor for CLADE, and many of the individuals in this study had more than 8 hours of screen exposure time. Therefore, to improve CLADE symptoms, it is necessary to propose a limit on screen time while wearing CL. Additionally, recent studies have demonstrated that hay fever and DED are pathologically related [42,43], thereby positing a synergistic effect of hay fever and DED on exacerbating CLADE symptoms. Moreover, our previous study demonstrated that hay fever, extended screen exposure time, and smoking are risk factors for severe dry eye symptoms [18]. Notably, these risk factors are modifiable and can be improved by lifestyle management [17]. Our findings would help identify individuals who are not yet diagnosed with CLADE and prevent deterioration of CLADE in routine clinical service and life.

Additionally, the types of CL were also identified using real-world data. Most of the CL users wore SCL and disposable lenses (Multimedia Appendix 5). However, the CL type and the daily CL duration did not correlate with CLADE (Table 2), as demonstrated in previous studies [44-46]. This study is a cross-sectional observational study; the causal relationship between CL type or daily duration of CL use and CLADE or CL discontinuation cannot be determined. Therefore, further study is needed to elucidate the associations between them.

Our study has several limitations associated with crowdsourced research, as presented in our previous studies [18,24,25]. First, this crowdsourced clinical study was characterized by selection bias for age, socioeconomic factors, and user characteristics because this app was released only for iOS (Apple Inc) devices. Furthermore, participants who were more interested in CLADE and had experienced CLADE symptoms might have completed all surveys and were subsequently included (Multimedia Appendix 3). Therefore, the prevalence of CLADE might have been overestimated. Second, this study might have recall bias because this study employed many self-administered questionnaires. We demonstrated the internal validity of the study [18]; however, considering the health-seeking behavior and cultural factors in Japan, the external validity or generalizability of the findings remains unknown. Additionally, socioeconomic status, education level, cultural background, and some important unmeasured factors related to CLADE were not investigated. In particular, this study found that one of the risk factors for CLADE was mental illness other than depression and schizophrenia, indicating that further precise classification for mental illness is needed. Further updates and development of an Android version of DryEyeRhythm and recruitment of individuals from other countries will reduce these biases. Third, this was a cross-sectional study; therefore, temporal relationships and causality between CLADE and the risk factors cannot be inferred. Additionally, this mobile health app study identified symptomatic dry eye based only on the OSDI questionnaire and might contain false-positives because dry eye examinations, including the Schirmer’s test and measurement of tear film break-up time, were not performed. However, this crowdsourced clinical study using DryEyeRhythm overcame several common participant recruitment–related issues, including diverse cohort and geographic restrictions, thus leading to the collection of real-world medical big data. Notably, it would be difficult to identify non-CLADE without our mobile app. Moreover, DryEyeRhythm also presents a unique opportunity for preventive care by identifying individuals at risk for CLADE earlier than currently possible. The app can be used to supplement traditional hospital-based research, thereby encompassing a broader population.

In conclusion, we identified individuals with CLADE and the associated risk factors using DryEyeRhythm. The various subjective symptoms of CLADE collected and stratified in this study could be used for the future prevention of CLADE.

## Appendix

Multimedia Appendix 1 Survey questions.

Multimedia Appendix 2 Daily subjective symptoms questions.

Multimedia Appendix 3 Sensitivity analysis between the included and excluded participants.

Multimedia Appendix 4 Characteristics of participants.

Multimedia Appendix 5 Characteristics of current contact lens users.

Multimedia Appendix 6 Age distributions of CL users.

## Abbreviations

CL: contact lens
CLD: contact lens discomfort
CLADE: contact lens–associated dry eye
DED: dry eye disease
OSDI: Ocular Surface Disease Index
SDS: Zung Self-Rating Depression Scale
t-SNE: t-distributed Stochastic Neighbor Embedding
