# Supplementary material for: Stratification of Individual Symptoms of Contact Lens–Associated Dry Eye Using the iPhone App DryEyeRhythm: Crowdsourced Cross-Sectional Study
Source: J Med Internet Res. 2020 Jun 26;22(6):e18996. doi: 10.2196/18996 (PMC7381048; doi:10.2196/18996)
Supplement: Multimedia Appendix 1 [file jmir_v22i6e18996_app1.doc]

**Table S1. Survey Questions.**

| **Questions** | **Variable Names** | **Variable Details** |
| --- | --- | --- |
|  | Age | Integer input, years |
|  | Sex | Choose one {"Man," "Woman"} |
|  | Height | Integer input, cm |
|  | Weight | Integer input, kg |
|  | Race | Choose one {"American Indian or Alaskan Native," "Arabic," "Asian," "Black or African American," "Caucasian," "Hispanic, Latino, or Spanish," "Native Hawaiian or other Pacific Islander," "Other Race"} |
| Have you ever been diagnosed with hypertension? | Hypertension | Choose one {"No," "I am being treated for hypertension," "I have untreated hypertension," "I do not know"} |
| Have you ever been diagnosed with diabetes? | Diabetes (HbA1c)^a^ | Choose one {"Yes," "No"}. If "Yes," scale bar input of HbA1c^a^ (5–15) |
| Have you suffered any of the following illnesses? | Systemic diseases | Multiple choices among {"Brain disease," "Blood disease," "Collagen disease," "Heart disease," "Kidney disease," "Liver disease," "Malignant tumor," "Respiratory disease," "N/A"^b^} |
| Do you have hay fever? | Hay fever | Choose one {"Yes," "No"} |
| Do you suffer from the following mental illnesses? | Mental illness | Multiple choice among {"Depression," "Schizophrenia," "Other mental illness," "N/A"^b^} |
| Have you ever been diagnosed as having dry eyes? | Past diagnosis of dry eye disease | Choose one {"Yes," "No"} |
| Have you ever undergone the following ophthalmic surgeries? | Ophthalmic surgery | Multiple choices among {"Cataract surgery," "LASIK,"^c^ "Other ophthalmic surgery," "N/A"^b^} |
| How much coffee did you drink today? | Coffee intake | Scale bar input (0–10), cups |
| Have you ever used contact lenses? | Contact lenses | Choose one {"I have been using contact lenses," "I have used contact lenses in the past," "I have not used contact lenses"} |
| What type of contact lens have you been using (or did you use?) | Contact lens type | Multiple choice among {"Soft contact lens/daily," "Soft contact lens/bi-weekly," "Soft contact lens/monthly," "Soft contact lens/conventional contact lenses," "Hard contact lens," "Color contact lens/daily," "Color contact lens/bi-weekly," "Color contact lens/monthly," "Color contact lens/conventional contact lenses"} |
| How long have you been using contact lenses | Contact lens term | Scale bar input (0 to more than 10), year |
| How long do you wear contact lenses a day? | Contact lens duration per day | Scale bar input (0–24), hours |
| Have you used eye drops? | Eye drop | Choose one {"Yes," "No"} |
| Please tell me how long you were exposed to the screen today (Smartphone, PC,^d^ TV,^e^ etc.). | Screen exposure time | Scale bar input (0–24), hours |
| How many hours do you exercise per week? | Periodic exercise | Scale bar input (0 to more than 10), hours |
| Please indicate at what time you went to bed. | Bedtime | Input drum bar, hours and minutes |
| Please indicate at what time you woke up this morning. | Wake-up time | Input drum bar, hours and minutes |
| Do you currently smoke, or have you smoked in the past? | Smoking | Choose one {"Yes," "No"} |
| How much fluid did you drink today? | Water intake | Scale bar input (0–2,000), mL |

^a^HbA1c=hemoglobin A1c; ^b^N/A=not applicable; ^c^LASIK=laser-assisted in situ keratomileusis; ^d^PC=personal computer; ^e^TV=television.
