# Supplementary material for: Stratification of Individual Symptoms of Contact Lens–Associated Dry Eye Using the iPhone App DryEyeRhythm: Crowdsourced Cross-Sectional Study
Source: J Med Internet Res. 2020 Jun 26;22(6):e18996. doi: 10.2196/18996 (PMC7381048; doi:10.2196/18996)
Supplement: Multimedia Appendix 2 [file jmir_v22i6e18996_app2.doc]

**Table S2. Daily Subjective Symptom Questions.**

| **Questions** | **Variable Names** | **Variable Details** |
| --- | --- | --- |
| Please answer each question using a 10-point scale | Stress level | Scale bar input (0, not at all to 10, I feel very stressed) |
|  | Headache | Scale bar input (0, not at all to 10, very painful) |
|  | Eye itching | Scale bar input (0, not at all to 10, very itchy) |
| Do you have any of the following symptoms that you aware of? | Concomitant symptoms | Multiple choices from {"Asthenopia," "Stiffness and pain of body axis muscle," "Mental fatigue," "N/A"^a^} |

^a^N/A=not applicable.
