# Supplementary material for: Stratification of Individual Symptoms of Contact Lens–Associated Dry Eye Using the iPhone App DryEyeRhythm: Crowdsourced Cross-Sectional Study
Source: J Med Internet Res. 2020 Jun 26;22(6):e18996. doi: 10.2196/18996 (PMC7381048; doi:10.2196/18996)
Supplement: Multimedia Appendix 3 [file jmir_v22i6e18996_app3.docx]

**Table S3. Sensitivity analysis between included and excluded participants.**

|  | **Included** | **Excluded** |  |  |
| --- | --- | --- | --- | --- |
|  | n = 4,454 (45.8) | n = 5,275 (54.2) | **Response number** | ***P* values** |
| CLADE^a^ (%) | 1,447 (78.5) | 1,600 (74.8) | 2,138 | .007 |
| Demographic characteristics |  |  |  |  |
| Age, median [IQR^b^], years | 23 [18-35] | 23 [18-33] | 5,272 | .03 |
| Age category (years), number (%) |  |  | 5,272 | .03 |
| <18 | 926 (20.8) | 1,138 (21.6) |  |  |
| 18–34 | 2,362 (53.0) | 2,891 (54.8) |  |  |
| 35–64 | 1,133 (25.4) | 1,204 (22.8) |  |  |
| ≥65 | 33 (0.7) | 39 (0.7) |  |  |
| Female, number (%) | 2,972 (66.7) | 3,090 (58.6) | 5,272 | <.001 |
| Height, median [IQR^b^], cm | 161 [156-168] | 163 [157-170] | 5,272 | <.001 |
| Body weight, median [IQR^b^], kg | 55 [49-65] | 56 [50-65] | 5,272 | .03 |
| Body mass index, median [IQR^b^] | 21.1 [19.2-23.5] | 21.0 [19.2-23.3] | 5,272 | .05 |
| Obesity (BMI^c^ ≥25), No. (%) | 725 (16.3) | 762 (14.5) | 5,272 | .01 |
| Medical history |  |  |  |  |
| Medicated hypertension, number (%) | 137 (3.1) | 103 (2.0) | 5,272 | <.001 |
| Diabetes, number (%) | 62 (1.4) | 54 (1.0) | 5,272 | .10 |
| Systemic diseases, number (%) |  |  |  |  |
| Blood disease | 32 (0.7) | 34 (0.6) |  | .66 |
| Brain disease | 31 (0.7) | 21 (0.4) | 5,272 | .05 |
| Collagen disease | 31 (0.5) | 19 (0.4) | 5,272 | .02 |
| Heart disease | 60 (1.6) | 74 (1.4) | 5,272 | .55 |
| Kidney disease | 60 (1.4) | 47 (0.9) | 5,272 | .03 |
| Liver disease | 45 (1.0) | 48 (0.9) | 5,272 | .61 |
| Malignant tumor | 27 (0.6) | 26 (0.5) | 5,272 | .45 |
| Respiratory disease | 276 (6.2) | 254 (4.8) | 5,272 | .003 |
| Hay fever, number (%) | 523 (45.1) | 591 (51.9) | 5.272 | .96 |
| Mental illness, number (%) |  |  |  |  |
| Depression | 181 (4.1) | 137 (2.6) | 5,272 | <.001 |
| Schizophrenia | 36 (0.8) | 32 (0.6) | 5,272 | .24 |
| Others | 210 (4.7) | 160 (3.0) | 5,272 | <.001 |
| Past diagnosis of dry eye disease, number (%) | 1,045 (23.5) | 1,160 (22.0) | 5,272 | .09 |
| Ophthalmic surgery, number (%) |  |  |  |  |
| Cataract surgery | 20 (0.5) | 22 (0.4) | 5,272 | .81 |
| LASIK | 62 (1.4) | 94 (1.8) | 5,272 | .13 |
| Others | 116 (2.6) | 135 (2.6) | 5,272 | .89 |
| Lifestyle Habits |  |  |  |  |
| Coffee, median [IQR^b^], cups per day | 0 [0-1] | 0 [0-1] | 1,128 | .03 |
| Eye drop use, number (%) | 877 (19.7) | 933 (17.7) | 5,272 | .01 |
| Contact lens use, number (%) |  |  | 5,272 | .35 |
| Negative | 2,146 (48.2) | 2,493 (47.3) |  |  |
| Current use | 1,844 (41.4) | 2,256 (42.8) |  |  |
| Past use | 464 (10.4) | 523 (10.0) |  |  |
| Screen exposure time, median [IQR^b^], hours per day | 6 [4-10] | 6 [4-10] | 1,128 | .09 |
| Screen exposure category, number (%), hours per day |  |  | 1,128 | .001 |
| <4 | 768 (20.0) | 220 (17.1) |  |  |
| 4–8 | 2,355 (55.0) | 597 (52.9) |  |  |
| >8 | 1,331 (25.0) | 311 (30.0) |  |  |
| Periodic exercise (positive vs. negative), number (%) | 2,919 (65.5) | 3,510 (66.6) | 5,272 | .28 |
| Periodic exercise, median [IQR^b^], hours per week | 1 [0-3] | 1 [0-3] | 5,272 | .01 |
| Sleeping time, median [IQR^b^], hours per day | 7 [6-8.5] | 7 [5.8-8.5] | 1,117 | .28 |
| Sleeping time category, number (%), hours per day |  |  | 1,117 | .15 |
| <6 | 1,282 (28.9) | 354 (31.7) |  |  |
| 6–9 | 2,355 (53.0) | 561 (50.2) |  |  |
| >9 | 804 (18.1) | 202 (18.1) |  |  |
| Smoking, number (%) | 1,060 (23.8) | 1,224 (23.2) | 5,272 | .50 |
| Water intake, median [IQR^b^], 100 mL per day | 8 [4-10] | 6 [4-10] | 1,128 | .008 |
| OSDI^e^ |  |  |  |  |
| OSDI^e^ total score, (0-100), median [IQR^b^] | 22.9 [12.5-36.1] | 20.8 [11.4-33.3] | 4,997 | <.001 |
| Normal (0-12), No. (%) | 1,160 (26.0) | 1,531 (30.6) |  | <.001 |
| Mild (13-22), No. (%) | 1,139 (25.6) | 1,307 (26.2) |  |  |
| Moderate (23-32), No. (%) | 778 (17.5) | 832 (16.7) |  |  |
| Severe (≥33), No. (%) | 1,377 (30.9) | 1,327 (26.6) |  |  |
| SDS^f^ |  |  |  |  |
| SDS^f^ total score, (20-80), median [IQR^b^] | 46 [39-54] | 47 [40-55] | 1,954 | .006 |
| Depressive symptom ≥40, number (%) | 3,271 (73.4) | 1,491 (76.3) | 1,954 | .002 |

^a^CLADE, contact lens-associated dry eye; ^b^IQR, interquartile range; ^c^BMI, body mass index; ^d^LASIK, laser-assisted in situ keratomileusis; ^e^OSDI, Ocular Surface Disease Index; ^f^SDS, Zung Self-rating Depression Scale.
