# Supplementary material for: Stratification of Individual Symptoms of Contact Lens–Associated Dry Eye Using the iPhone App DryEyeRhythm: Crowdsourced Cross-Sectional Study
Source: J Med Internet Res. 2020 Jun 26;22(6):e18996. doi: 10.2196/18996 (PMC7381048; doi:10.2196/18996)
Supplement: Multimedia Appendix 4 [file jmir_v22i6e18996_app4.docx]

**Table S4. Characteristics of participants.**

| Characteristic | | | | | | Contact lens use | | | *P* value | Total |
| --- | --- | --- | --- | --- | --- | --- | --- | --- | --- | --- |
|  | | | | | | Negative | Past use | Current use |  |  |
|  | | | | | | n=2146 (48.2%) | n=464 (10.4%) | n=1844 (41.4%) |  | N=4454 (100%) |
|  | | | | | |  |  |  |  |  |
| Age (years), median (IQR) | | | | | | 23 (18-40) | 33 (24-44) | 22 (18-28) | <.001 | 23 (18-35) |
| **Age category (years), n (%)** | | | | | |  |  |  | <.001 |  |
|  | | | | | <18 | 535 (24.9) | 31 (6.7) | 360 (19.5) |  | 926 (20.8) |
|  | | | | | 18-34 | 943 (43.9) | 213 (45.9) | 1206 (65.4) |  | 2362 (53.0) |
|  | | | | | 35-64 | 637 (29.7) | 219 (47.2) | 277 (15.0) |  | 1133 (25.4) |
|  | | | | | ≥65 | 31 (1.4) | 1 (0.2) | 1 (0.1) |  | 33 (0.7) |
| Female, n (%) | | | | | | 1236 (57.6) | 265 (57.1) | 1471 (79.8) | <.001 | 2972 (66.7) |
| Height (cm), median (IQR) | | | | | | 162 (157-170) | 163 (157-171) | 160 (156-165) | <.001 | 161 (156-168) |
| Body weight (kg), median (IQR) | | | | | | 57 (50-66) | 60 (52-68) | 53 (48-60) | <.001 | 55 (49-65) |
| Body mass index, median (IQR) | | | | | | 21.4 (19.5-24.1) | 22.0 (20.0-24.4) | 20.7 (19.1-22.7) | <.001 | 21.1 (19.2-23.5) |
| Obesity (BMI ≥25), n (%) | | | | | | 419 (19.5) | 100 (21.6) | 206 (11.2) | <.001 | 725 (16.3) |
| Medicated hypertension, n (%) | | | | | | 88 (4.1) | 23 (5.0) | 26 (1.4) | <.001 | 137 (3.1) |
| Diabetes, n (%) | | | | | | 42 (2.0) | 11 (2.4) | 9 (0.5) | <.001 | 62 (1.4) |
| **Systemic disease, n (%)** | | | | | |  |  |  |  |  |
|  | | | | | Blood disease | 18 (0.8) | 6 (1.3) | 8 (0.4) | .10 | 32 (0.7) |
|  | | | | | Brain disease | 17 (0.8) | 7 (1.5) | 7 (0.4) | .03 | 31 (0.7) |
|  | | | | | Collagen disease | 15 (0.7) | 7 (1.5) | 9 (0.5) | .06 | 31 (0.7) |
|  | | | | | Heart disease | 38 (1.8) | 5 (1.1) | 26 (1.4) | .45 | 69 (1.6) |
|  | | | | | Kidney disease | 29 (1.4) | 23 (1.3) | 8 (1.7) | .73 | 60 (1.4) |
|  | | | | | Liver disease | 23 (1.1) | 9 (1.9) | 13 (0.7) | .06 | 45 (1.0) |
|  | | | | | Malignant tumor | 14 (0.7) | 3 (0.7) | 10 (0.5) | .90 | 27 (0.6) |
|  | | | | | Respiratory disease | 119 (5.6) | 42 (9.1) | 115 (6.2) | .02 | 276 (6.2) |
| Hay fever, n (%) | | | | | | 1077 (50.2) | 239 (51.5) | 933 (50.6) | .87 | 2249 (50.5) |
| **Mental illness, n (%)** | | | | | |  |  |  |  |  |
|  | | Depression | | | | 98 (4.6) | 28 (6.0) | 55 (3.0) | .003 | 181 (4.1) |
|  | | Schizophrenia | | | | 18 (0.8) | 4 (0.9) | 14 (0.8) | .95 | 36 (0.8) |
|  | | Others | | | | 92 (4.3) | 34 (7.3) | 84 (4.6) | .02 | 210 (4.7) |
| Past diagnosis of dry eye disease, n (%) | | | | | | 386 (18.0) | 178 (26.1) | 481 (26.1) | <.001 | 1045 (23.5) |
| **Ophthalmic surgery, n (%)** | | | | | |  |  |  |  |  |
|  | | Cataract surgery | | | | 15 (0.7) | 5 (1.1) | 0 (0) | <.001 | 20 (0.5) |
|  | | LASIK^a^ | | | | 16 (0.8) | 42 (9.1) | 4 (0.2) | <.001 | 62 (1.4) |
|  | | Others | | | | 50 (2.3) | 25 (5.4) | 41 (2.2) | <.001 | 116 (2.6) |
| **Lifestyle habits** | | | | | |  |  |  |  |  |
|  | | Coffee (cups per day), median (IQR) | | | | 0 (0-2) | 1 (0-2) | 0 (0-1) | <.001 | 0 (0-1) |
|  | | Eye drop use, n (%) | | | | 288 (13.4) | 109 (23.5) | 480 (26.0) | <.001 | 877 (19.7) |
|  | | Screen exposure time (h/d)^b^, median (IQR) | | | | 6 (4-10) | 6 (4-10) | 6 (4-10) | .003 | 6 (4-10) |
| **Screen exposure category (h/d), n (%)** | | | | | |  |  |  | .65 |  |
|  | | <4 | | | | 380 (17.7) | 73 (15.7) | 315 (17.1) |  | 768 (17.2) |
|  | | 4-8 | | | | 1143 (53.3) | 242 (52.2) | 970 (52.6) |  | 2355 (52.9) |
|  | | >8 | | | | 623 (29.0) | 149 (32.1) | 559 (30.3) |  | 1331 (29.9) |
| Periodic exercise (positive vs negative), n (%) | | | | | | 1483 (69.1) | 270 (58.2) | 1166 (63.2) | <.001 | 2919 (65.5) |
| Periodic exercise (h/wk)^c^, median (IQR) | | | | | | 1 (0-4) | 1 (0-3) | 1 (0-3) | <.001 | 1 (0-3) |
| Sleeping time (h/d), median (IQR) | | | | | | 7 (6.0-8.5) | 7 (6.0-8.3) | 7 (6.0-8.5) | <.001 | 7 (6.0-8.5) |
| **Sleeping time category (h/d), n (%)** | | | | | |  |  |  | .13 |  |
|  | <6 | | | | | 1124 (52.4) | 269 (58.0) | 962 (52.2) |  | 2355 (52.9) |
|  | 6-9 | | | | | 620 (28.9) | 128 (27.6) | 534 (29.0) |  | 1282 (28.8) |
|  | >9 | | | | | 402 (18.7) | 67 (14.4) | 348 (18.9) |  | 817 (18.3) |
| Smoking, n (%) | | | | | | 493 (23.0) | 164 (35.3) | 401 (21.8) | <.001 | 1058 (23.8) |
| Water intake (100 mL/d), median (IQR) | | | | | | 8 (4-10) | 8 (4-10) | 8 (4-10) | .22 | 8 (4-10) |
| **OSDI^d^ score** | | | | | |  |  |  |  |  |
|  | | | | OSDI total score (0-100), median (IQR) | | 20.8 (11.4-33.3) | 22.9 (12.5-36.7) | 25 (14.6-39.6) | .05 | 22.9 (12.5-36.1) |
|  | | | | Normal (0-12), n (%) | | 633 (29.5) | 130 (28.0) | 397 (21.5) | <.001 | 1160 (26.0) |
|  | | | | Mild (13-22), n (%) | | 587 (27.3) | 106 (22.8) | 446 (24.2) |  | 1139 (25.6) |
|  | | | | Moderate (23-32), n (%) | | 340 (15.8) | 78 (16.8) | 360 (19.5) |  | 778 (17.5) |
|  | | | | Severe (≥33), n (%) | | 586 (27.3) | 150 (32.3) | 641 (34.8) |  | 1377 (30.9) |
| **SDS^e^ score** | | | | | |  |  |  |  |  |
|  | | | SDS total score (20-80), median (IQR) | | | 46 (39-53) | 46 (38-52) | 46 (40-54) | .59 | 46 (39-54) |
|  | | | SDS score ≥40, n (%) | | | 1547 (72.1) | 331 (71.3) | 1393 (75.5) | .03 | 3271 (73.4) |

^a^LASIK: laser-assisted in situ keratomileusis.

^b^h/d: hours per day.

^c^h/wk: hours per week.

^d^OSDI: Ocular Surface Disease Index (Allergan Inc).

^e^SDS: Zung Self-Rating Depression Scale.
