# Supplementary material for: Stratification of Individual Symptoms of Contact Lens–Associated Dry Eye Using the iPhone App DryEyeRhythm: Crowdsourced Cross-Sectional Study
Source: J Med Internet Res. 2020 Jun 26;22(6):e18996. doi: 10.2196/18996 (PMC7381048; doi:10.2196/18996)
Supplement: Multimedia Appendix 5 [file jmir_v22i6e18996_app5.docx]

Table S5. Characteristics of current contact lens users.

| Characteristic | | | Non-CLADE^a^ | CLADE | *P* values | Total |
| --- | --- | --- | --- | --- | --- | --- |
|  | | | n=397 (21.5%) | n=1447 (78.5%) |  | N=1844 (100%) |
|  | | |  |  |  |  |
| Age (years), median (IQR) | | | 23 (18-31) | 22 (18-28) | .08 | 22 (18-28) |
| **Age category, n (%)** | | |  |  | .07 |  |
|  | <18 | | 74 (18.6) | 286 (19.8) |  | 360 (19.5) |
|  | 18-34 | | 251 (63.2) | 955 (66.0) |  | 1206 (65.4) |
|  | 35-64 | | 71 (17.9) | 206 (14.2) |  | 277 (15.0) |
|  | ≥65 | | 1 (0.3) | 0 (0) |  | 1 (0.05) |
| Female, n (%) | | | 287 (72.3) | 1184 (81.8) | <.001 | 1471 (79.8) |
| Height (cm), median (IQR) | | | 161 (156-167) | 160 (156-165) | .003 | 160 (156-165) |
| Body weight (kg), median (IQR) | | | 54 (49-63) | 53 (48-60) | .01 | 53 (48-60) |
| Body mass index, median (IQR) | | | 20.8 (19.1-22.8) | 20.6 (19.1-22.7) | .45 | 20.7 (19.1-22.7) |
| Obesity (BMI ≥25), n (%) | | | 40 (10.1) | 166 (11.5) | .43 | 206 (11.2) |
| CL^b^ use (years), median (IQR) | | | 6 (3-10) | 6 (3-10) | .37 | 6 (3-10) |
| CL duration (h/d)^c^, median (IQR) | | | 14 (12-16) | 14 (12-16) | .15 | 14 (12-16) |
| **CL duration (h/d), n (%)** | | |  |  | .33 |  |
|  | 0-6 | | 25 (6.3) | 74 (5.1) |  | 99 (5.4) |
|  | 6-12 | | 132 (33.3) | 490 (33.9) |  | 622 (33.7) |
|  | 12-18 | | 218 (54.9) | 768 (53.1) |  | 986 (53.5) |
|  | 18-24 | | 22 (5.5) | 115 (8.0) |  | 137 (7.4) |
| **Contact lens type, n (%)** | | |  |  | .89 |  |
|  | Hard contact lens | | 25 (6.3) | 94 (6.5) |  | 119 (6.45) |
|  | Soft contact lens | | 372 (93.7) | 1353 (93.5) |  | 1725 (93.6) |
| **Disposable lens (noncolored), n (%)** | | |  |  |  |  |
|  | Daily | | 179 (45.1) | 644 (44.5) | .836 | 823 (44.6) |
|  | Biweekly^d^ | | 179 (45.1) | 562 (38.8) | .024 | 741 (40.2) |
|  | Monthly | | 20 (5.0) | 60 (4.2) | .440 | 80 (4.3) |
| Conventional lens (noncolored), n (%) | | | 13 (3.3) | 41 (2.8) | .644 | 54 (2.9) |
| **Disposable lens (colored), n (%)** | | |  |  |  |  |
|  | | Daily | 36 (9.1) | 220 (15.2) | .002 | 256 (13.4) |
|  | | Biweekly^d^ | 8 (2.0) | 59 (4.1) | .052 | 67 (3.6) |
|  | | Monthly | 12 (3.0) | 99 (6.8) | .005 | 111 (6.0) |
| Conventional lens (colored), n (%) | | | 6 (1.5) | 57 (3.9) | .018 | 63 (3.4) |
| Medicated hypertension, n (%) | | | 9 (2.3) | 17 (1.2) | .11 | 26 (1.4) |
| Diabetes, n (%) | | | 2 (0.5) | 7 (0.5) | .96 | 9 (0.5) |
| **Systemic disease, n (%)** | | |  |  |  |  |
|  | | Blood disease | 1 (0.3) | 7 (0.5) | .53 | 8 (0.4) |
|  | | Brain disease | 2 (0.5) | 5 (0.4) | .65 | 7 (0.4) |
|  | | Collagen disease | 1 (0.3) | 8 (0.6) | .45 | 9 (0.5) |
|  | | Heart disease | 3 (0.8) | 23 (1.6) | .21 | 26 (1.4) |
|  | | Kidney disease | 2 (0.5) | 21 (1.5) | .13 | 23 (1.3) |
|  | | Liver disease | 2 (0.5) | 11 (0.8) | .59 | 13 (0.7) |
|  | | Malignant tumor | 0 (0) | 10 (0.7) | .10 | 10 (0.5) |
|  | | Respiratory disease | 17 (4.3) | 98 (6.8) | .07 | 115 (6.2) |
| Hay fever, n (%) | | | 174 (43.8) | 759 (52.5) | .002 | 933 (50.6) |
| **Mental illness, n (%)** | | |  |  |  |  |
|  | | Depression | 7 (1.8) | 48 (3.3) | .11 | 55 (3.0) |
|  | | Schizophrenia | 3 (0.8) | 11 (0.8) | .99 | 14 (0.8) |
|  | | Other | 7 (1.8) | 77 (5.3) | .003 | 84 (4.6) |
| Past diagnosis of dry eye disease, n (%) | | | 64 (16.1) | 417 (28.8) | <.001 | 481 (26.1) |
| **Ophthalmic surgery, n (%)** | | |  |  |  |  |
|  | | Cataract surgery | 0 (0) | 0 (0) | N/A^e^ | 0 (0) |
|  | | LASIK^f^ | 2 (0.5) | 2 (0.1) | .17 | 4 (0.2) |
|  | | Other | 4 (1.0) | 37 (2.6) | .06 | 41 (2.2) |
| **Lifestyle habits** | | |  |  |  |  |
|  | | Coffee (cups per day), median (IQR) | 0 (0-1) | 0 (0-1) | .872 | 0 (0-1) |
|  | | Eye drop use, n (%) | 76 (19.1) | 404 (27.9) | <.001 | 480 (26.0) |
|  | | Screen exposure time (h/d), median (IQR) | 6 (4-8) | 6 (4-10) | <.001 | 6 (4-10) |
| **Screen exposure category (h/d), n (%)** | | |  |  | .01 |  |
|  | | <4 | 77 (19.4) | 238 (16.5) |  | 315 (17.1) |
|  | | 4-8 | 224 (56.4) | 746 (51.2) |  | 970 (52.6) |
|  | | >8 | 96 (24.2) | 463 (32.0) |  | 559 (30.3) |
| Periodic exercise (positive vs negative), n (%) | | | 264 (66.5) | 902 (62.3) | .13 | 1166 (63.2) |
| Periodic exercise (h/wk)^g^, median (IQR) | | | 1 (0-4) | 1 (0-3) | .08 | 1 (0-3) |
| Sleeping time (h/d), median (IQR) | | | 7 (6-8.3) | 7 (6-8.7) | .19 | 7 (6-8.5) |
| **Sleeping time category (h/d), n (%)** | | |  |  | .40 |  |
|  | | <6 | 216 (54.4) | 746 (51.6) |  | 962 (52.2) |
|  | | 6-9 | 115 (29.0) | 419 (29.0) |  | 534 (29.0) |
|  | | >9 | 66 (16.7) | 282 (19.5) |  | 348 (18.9) |
| Smoking, n (%) | | | 61 (15.4) | 340 (23.5) | .001 | 401 (21.8) |
| Water intake (100 mL/d), median (IQR) | | | 8 (4-10) | 8 (4-10) | .50 | 8 (4-10) |

^a^CLADE: contact lens–associated dry eye.

^b^CL: contact lens.

^c^h/d: hours per day.

^d^Biweekly: every 2 weeks.

^e^N/A: not applicable.

^f^LASIK: laser-assisted in situ keratomileusis.

^g^h/wk: hours per week.
