# Supplementary material for: Stratification of Individual Symptoms of Contact Lens–Associated Dry Eye Using the iPhone App DryEyeRhythm: Crowdsourced Cross-Sectional Study
Source: J Med Internet Res. 2020 Jun 26;22(6):e18996. doi: 10.2196/18996 (PMC7381048; doi:10.2196/18996)
Supplement: Multimedia Appendix 6 [file jmir_v22i6e18996_app6.doc]

**Figure S1.** Age distributions of CL users. The frequency of current CL use was significantly decreased with aging (β=-0.008; 95% CI, -0.009 to -0.007; *P*<.001). Conversely, the frequency of past CL use was significantly increased with aging (β=0.004; 95% CI, 0.004 to 0.005; *P*<.001). CL, contact lens; CI, confidence interval
